# Radiotherapy and Chemotherapy Features in the Treatment for Locoregional Recurrence of Endometrial Cancer: A Systematic Review

**DOI:** 10.3390/jpm13060886

**Published:** 2023-05-24

**Authors:** Carlo Ronsini, Irene Iavarone, Antonella Reino, Maria Giovanna Vastarella, Pasquale De Franciscis, Angelo Sangiovanni, Luigi Della Corte

**Affiliations:** 1 Department of Woman, Child and General and Specialized Surgery, University of Campania Luigi Vanvitelli, 80138 Naples, Italy; ireneiavarone2@gmail.com (I.I.); antonella.reino.11@gmail.com (A.R.); mariagiovanna.vastarella@studenti.unicampania.it (M.G.V.); pasquale.defranciscis@unicampania.it (P.D.F.); 2 Department of Precision Medicine, University of Campania Luigi Vanvitelli, 80138 Naples, Italy; angelo.sangiovanni@unicampania.it; 3 Department of Neuroscience, Reproductive Sciences and Dentistry, School of Medicine, University of Naples Federico II, 80131 Naples, Italy; luigi.dellacorte@unina.it

**Keywords:** endometrial cancer, radiotherapy, recurrence, locoregional, brachytherapy

## Abstract

Radiation therapy (RT) is the standard of care in patients with locoregional or isolated vaginal recurrence who never underwent irradiation. It is often associated with brachytherapy (BT), whereas chemotherapy (CT) is a rare treatment option. We systematically searched the PubMed and Scopus databases in February 2023. We included patients with relapsed endometrial cancer, describing the treatment of locoregional recurrence, and reporting at least one outcome of interest—disease-free survival (DFS), overall survival (OS), recurrence rate (RR), site of recurrence, and major complications. A total of 15 studies fulfilled the inclusion criteria. Overall, 11 evaluated RT only, 3 evaluated CT, and 1 analyzed oncological outcomes after administration with a combination of CT and RT. In total, 4.5-year OS ranged from 16% to 96%, and DFS ranged from 36.3% to 100% at 4.5 years. RR ranged from 3.7% to 98.2% during a median follow-up of 51.5 months. Overall, RT showed a 4.5-year DFS from 40% to 100%. CT revealed 36.3% DFS at 4.5 years. RT showed a 4.5-year OS ranging from 16% to 96%, whereas CT revealed a 27.7% OS rate. It would be appropriate to test multi-modality regimens to evaluate outcomes and toxicity. EBRT and BT are the most employed options to treat vaginal recurrences.

## 1. Introduction

Endometrial cancer is the most common gynecologic cancer [1]. Most patients have diseases confined to the uterine corpus and are treated surgically [1]. Leading scientific societies have stratified patients into four risk classes [2]. Patients at low and intermediate risk are associated with a low risk of recurrence, while patients at intermediate-high and high risk have a risk of relapse of up to 15% and 26% [1,3,4,5]. Approximately 75% of these recurrences are isolated to the vaginal cuff [6,7,8]. Currently, there is a lack of standards for diagnosing and treating this type of recurrence. The three main approaches involve surgery, chemotherapy, and radiotherapy. Radiotherapy, in turn, can be either external beam (EBRT) or brachytherapy (BRT). Radiation therapy (RT) is currently a widely diffused treatment of choice in previously unirradiated patients with locoregional or isolated vaginal recurrence [9,10,11]. In previously irradiated patients, radical surgery, including pelvic exenteration, is considered [12,13,14]. It is also possible to consider RT associated or not with systemic therapy as a radical treatment option [15]. The interstitial BT with or without EBRT can result in high local disease control at 1–5 years [16,17]. Little data exist on the exclusive use of systemic therapies [18]. Although it is well known that combining chemotherapeutic agents is more effective than administering one chemotherapeutic drug only [18,19,20]. Despite the increased survival outcomes, poly-CT is linked to a higher rate of toxic reactions [20,21]. Given the frequency of this type of recurrence, the risk-benefit ratio of individual approaches needs to be clarified. Since the pathology is limited to the vaginal dome, systemic treatments are likely to have unmotivated sequelae for achieving pathology control, compared to BRT [22,23]. This study aimed to analyze and compare treatment options for locoregional or vaginal recurrence in endometrial cancer, focusing on oncological outcomes and their comorbidities.

## 2. Materials and Methods

The methods for this study were specified a priori based on the recommendations in the Preferred Reporting Items for Systematic Reviews and Meta-Analyses (PRISMA) statement [24]. The present study was registered on PROSPERO as ID409473.

### 2.1. Search Method

We performed systematic research for records about therapies used to treat endometrial cancer with isolated locoregional recurrence in PubMed, EMBASE, and Scopus in February 2023. We did not restrict the country or the year of publication and considered only studies published entirely in English. We adopted the following string of idioms in each database to identify studies fitting our review’s topic: “Neoplasm Recurrence, Local AND therapy AND “Endometrial Neoplasms”.

### 2.2. Study Selection

**Figure 1:**
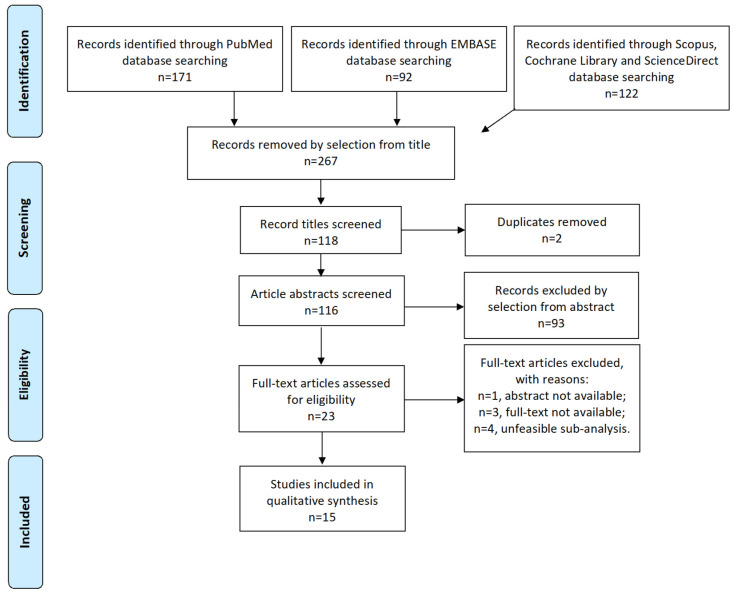
Preferred Reporting Items for Systematic Reviews and Meta-Analyses (PRISMA) Flow-diagram.

Study selection was made independently by I.I. and A.R. In case of discrepancy, C.R. decided on inclusion or exclusion. Inclusion criteria were: (1) studies including patients treated for isolated locoregional recurrence of endometrial cancer; (2) studies reporting at least one outcome of interest: disease-free survival (DFS), overall survival (OS), recurrence rate (RR), site of recurrence, and major complications according to Clavien–Dindo Classification [23]; and (3) peer-reviewed articles, published originally. We excluded: non-original studies, pre-clinical trials, animal trials, abstract-only publications, and articles in a language other than English. If possible, the authors of studies that were published as conference abstracts were contacted via e-mail and asked to provide their data. We mentioned the studies selected and all reasons for exclusion in the Preferred Reporting Items for Systematic Reviews and Meta-Analyses (PRISMA) flow-chart (Figure 1). We assessed all included studies concerning potential conflicts of interest.

### 2.3. Data Extraction

I.I. and A.R. extracted data from all relevant series concerning tumor characteristics, surgical approach, morbidity, and oncological issues such as Recurrences, Deaths, Recurrence Rate (RR), Disease-free Survival (DFS), Overall Survival (OS), and Upstaging rate. Additionally, the two groups extracted and compared data about perioperative complications (graded according to the Clavien–Dindo scale) [25]. Disease-free survival was defined as the time elapsed between surgery and recurrence. Overall survival was considered as the time elapsed between surgery and death for disease or the last follow-up. Cancer Recurrence was referred to the detection of disease after treatment and after a period of time when the tumor could not be found. The recurrence rate (RR) was the percentage of patients from each study that showed cancer recurrence.

## 3. Results

### 3.1. Studies’ Characteristics

After the database search, 267 articles matched the search criteria. After removing records with no full text available, duplicates, abstracts unfitting to the topic of our review, and wrong study designs (e.g., reviews), 23 were suitable for eligibility. Of those, 15 matched the inclusion criteria and were included in the systematic review. In total, 11 of them were non-comparative, single-armed studies evaluating RT only. Three of them were single-arm studies evaluating chemotherapy (CT), whereas one study analyzed oncological outcomes after administration with a combination of chemotherapeutic agents and RT (Table 1). The countries where the studies were conducted, the studies’ design, the enrollment year range, FIGO stage of disease, the number of participants, treatments used, and follow-up (FU) time are summarized in Table 1. Overall, the publication years ranged from 1980 to 2019. In total, 3205 patients with LRR recurrence of EC, treated with RT, were included in the systematic review. FU periods ranged from 27 to 102 months on average. A total of 3205 patients were included in the review. Of the 15 selected studies, 14 presented DFS and OS data. Except for 3, the other 12 studies presented RR data. The 4.5-year OS ranged from 16% to 96%. The 4.5-year DFS ranged from 36.3% to 100%. The 3-year OS ranged from 21% to 80%. The 3-year DFS ranged from 16.6% to 86%. RR ranged from 3.7% to 98.2%. Overall, the RT was administered with an intensity between 45 Gy and 81 Gy. Three studies reported high-dose regimens, with an administration of a cumulative equivalent dose of 45 to 75.5 Gy, divided into 2-Gy fractions.

### 3.2. Disease-Free Survival Analysis

Regarding DFS, Francis et al. showed the highest 4.5-year DFS of 100% in a cohort of patients administered with salvage EBRT [37]. Those data were followed by 4.5-year DFS in the analysis by Petignat et al., detecting 96% DFS in women treated with high-dose-rate interstitial BT [31]. In the study by Chapman et al., 4.5-year DFS was 86% after salvage high-dose-rate interstitial BT and EBRT [36]. In addition, the same cohort revealed the highest DFS rate at 3 years, e.g., 86% [36]. Similarly, Nag et al. and Huh et al. revealed 85.8% and 83% 4.5-year DFS in patients administered with EBRT +/− BT [29,32]. In the analysis performed by Vance et al., 77% of patients were disease-free at 4–5 years and were also administered with EBRT +/− BT [35]. Lower DFS rates were detected at 4.5 years in the studies conducted by Wylie et al., Sears et al., and Kuten et al. in 68%, 54%, and 40% of patients, respectively, who were administered with EBRT +/− BT [27,28,30]. The cohort of Attarian et al. showed the lowest 4.5-year DFS of 36% after administration with carboplatin and Paclitaxel [33]. Those results are summarized in Table 2. Overall, RT showed a 4.5-year DFS ranging from 40% to 100%. CT revealed 36.3% DFS at 4.5 years. At 3 years, RT alone showed DFS rates from 40% to 86%, whereas CT revealed DFS from 16.6% alone to 35.2% in combination with RT.

### 3.3. Overall Survival Analysis

Regarding OS, the highest 4.5-year rates were shown by Petignat et al. with 96% OS [31]. Secondarily, Chapman et al., Huh et al., and Vance et al. showed 4.5-year OS rates of 77%, 75%, and 72%, respectively, and their cohorts were treated with EBRT +/− BT [32,35,36]. Francis et al. showed 65% OS at 4.5 years in patients administered with salvage EBRT [37]. The lowest 4.5-year OS rates were found in the articles written by Attarian et al., Kuten et al., and Pirtoli et al., and they were 27%, 18%, and 16%, respectively [26,27,33]. The former was based on carboplatin and Paclitaxel administration, whereas the cohorts pf Kuten et al. and Pirtoli et al. were treated with EBRT +/− RT [26,27,33]. Overall, RT showed a 4.5-year OS ranging from 16% to 96%. CT revealed 27.7% OS at 4.5 years. At 3 years, RT showed OS rates from 21% to 80%, whereas CT alone revealed OS from 50% to 80%. Those data are also visible in Table 2.

### 3.4. Recurrence Patterns

Regarding recurrence, in the retrospective analysis conducted by Francis et al., only 3.7% of patients administered with salvage EBRT recurred in a mean FU time of 74 months [37]. In the study by Petignat et al., the recurrence rate corresponded to 4.5% in women treated with high-dose-rate interstitial BT during a FU time of 32 months on average [26]. The cohorts of Chapman et al., Huh et al., Vance et al., and Sears et al. showed a RR of 14%, 17%, 23%, and 23%, respectively, and patients were treated with EBRT +/− BT during FU period ranging from 89-to-56 months on average [28,32,35,36]. The highest RRs were found in the articles written by Wylie et al., Kuten et al., and Kamran et al. and corresponded to 98.2%, 76.4%, and 62%, respectively [17,27,30]. Patients were administered with doxorubicin, 5-fluorouracil, etoposide, and cisplatin in the study conducted by Wylie et al., EBRT +/− BT in the study by Kuten et al., and high-dose-rate interstitial BT in the article by Kamran et al. [17,27,30]. RR data are summarized in Table 2.

In 5 of them, extracting data about sites of recurrence and major complications was also feasible, as shown in Table 3 and Table 4. In 76.4% of patients with recurrence after BT in Kuten et al. study, the site of recurrence was the pelvis [27]. Local recurrence corresponded to 13.7% [27]. Moreover, in a study by Wylie et al., recurrence sites in 98.2% of women were the vagina and pelvis after EBRT [30]. In that context, local recurrence corresponded to 6.8% [30]. Additionally, in the article by Kamran et al., the recurrence of the disease was detected in the vagina [17]. In studies by both Nag et al. and Petignat et al., EC recurred in para-aortic nodes after EBRT +/− BT and high-dose-rate interstitial BT, respectively [29,31]. Recurrence in visceral organs and cavities such as the lung, liver, mediastinum and the perihepatic region occurred in the analyses of Nag et al. and Kamran et al. [17,29]. In addition, Petignat et al. and Kamran et al. detected a recurrence of disease in the bone. Only Kamran et al. detected recurrence in the iliac, inguinal, and hilar nodes and into the abdominal cavity and peritoneum after high-dose-rate interstitial BT in both cases [17,31]. In the study by Kamran et al., local recurrence corresponded to 10.6%, distant recurrence was 21.2%, and mixed recurrence was 4.5% [17]. Those results are summarized in Table 3.

### 3.5. Complication Rate

Regarding the complication rate, Kuten et al. detected 11.7% of major complications according to Clavien–Dindo Classification, including small bowel obstruction, ureteral obstruction, vaginal vault stenosis, and osteonecrosis after EBRT +/− BT [27]. Moreover, Nag et al. found 14.2% of severe Clavien–Dindo complications, such as vaginal stenosis, in women administered with the same modalities [29]. The studies by Pierga et al. and Attarian et al. were the only which detected no recurrence in patients administered with a combination of doxorubicin, 5-fluorouracil, etoposide, and cisplatin CT and carboplatin and Paclitaxel chemotherapy, respectively [18,33]. Smaniotto et al. presented 13.3% of severe complications combining chemotherapeutic agents and EBRT +/− BT [15]. Petignat et al. had the highest grade 3 complication rate, involving 68.1% of patients administered with high-dose-rate interstitial BT [26], followed by Leslie et al., who detected 46.6% of complications in women treated with Lapatinib [34]. Kamran et al. detected 34% of complications, primarily urinary and rectal [17]. The only record employing RT regimens with a low rate of grade 3 or more Clavien–Dindo complications was the study by Huh et al., with a 5% complication rate [32]. The present data are summarized in Table 4.

## 4. Discussion

The treatment for the recurrence of EC is heterogeneous because it reflects various manifestations of recurrence. Locoregional recurrence—considered the involvement of the vaginal vault only—represents the most common manifestation among recurrences in EC [6,37,38]. The pathophysiological reasons underlying that phenomenon are not currently clear. They could be attributed to the appropriateness of surgical techniques, which, similarly to what has been seen for cervical cancer, could spread carcinomatous cells into the surgical field [39,40,41]. They could also be attributed to molecular mechanisms of tumor instability and aggressiveness [39,40,42]. The tumor microenvironment, such as lymphovascular space invasion (LVSI), may also play a role in the recurrence of EC [43,44]. Finally, a confounding factor could be the appropriateness of surgical staging [39,42,45,46]. The diffusion of sentinel lymph node mapping and progressive abandonment of systematic lymphadenectomy in EC can commonly lead to under-staging [43,47]. In our opinion, that risk can only partially justify locoregional recurrence and should be related to lymph nodes or distant recurrence.

However, it could still influence the choice of adjuvant treatment and therefore minimize the adequacy of the offer [48]. The isolated nature of locoregional recurrence may enlighten surgery as a valid and resolutive approach. Unfortunately, data regarding the use of surgery to treat locoregional recurrences in EC need to be more present. Nonetheless, demolitive options range from colpectomy to pelvectomy with generally increasing morbidity and quality of life impact [49,50]. For that reason, in previously unirradiated patients, the main approach is RT because it guarantees local control and minimizes morbidity [27,29,32]. However, the main difficulty in managing those patients is identifying vaginal vault recurrences with no microscopic diffusion that would risk insufficient treatment. Therefore, our review also focused on the site of the second recurrence. The hypothetically adequate treatments for local control should show minimum rates of second locoregional recurrences, whilst only distant recurrences should occur (in previously untreated regions). CT-based regimens have shown a lower risk of second distant recurrences [18,33,34].

Regarding multimodality treatment options, Smaniotto et al. combined multi-agent CT, RT, with or without a boost of BT or EBRT and evaluated the outcomes of their cohort based on prognostic factors of worse local control: short time between surgery and recurrence, low hemoglobin concentration, recurrence on the pelvic wall, and positivity of lymph nodes [15]. The authors detected better local control and better outcomes in patients with the absence of those characteristics, enhancing the potential need for a boost of EBRT or BT in patients with predicting factors of worse local control [15]. In those cases, the rate of complications requiring surgery after multimodality treatment—according to Clavien–Dindo classification—was 13.3%, which is lower compared to other articles assessing RT and/or BT alone [15]. Unfortunately, the other records assessing multi-agent CT do not provide data about toxicity [18,33], whereas, in the study by Leslie et al., single-agent CT with Lapatinib shows 46.6% of complications [34].

Among prognostic factors, it would be useful to consider the stage of the disease. Studies analyzing patients with stage I-II of EC showed better DFS and OS outcomes at 3 years and 4.5 years after the treatment of locoregional recurrences [30,31,32,35,37]. Otherwise, results concerning secondary recurrence rates and sites of recurrence are heterogeneous. For example, Wylie et al. treated patients with EBRT only, revealing 68% 4.5-year DFS and 53% 4.5-year OS [30]. RR corresponded to 98.2% in the vagina and pelvis [30]. Petignat et al. administered high-dose-rate interstitial BT, and they obtained the highest DFS (96%) and OS (96%) at 4.5 years [31]. Moreover, in the present cohort, RR was low (4.5%), but sites of recurrence were para-aortic nodes and bone [31]. It is difficult to determine which regimen could guarantee local and distant disease control in locoregional recurrences of EC due to the heterogeneity of data. In Huh et al., Vance et al., and Francis et al., there is no information about the site of recurrence. Huh et al. and Vance et al.—compared to Francis et al.—present higher OS rates at 4.5 years (75% and 72%, respectively), with RR of 17% and 23%, respectively, after administrating patients with EBRT and/or BT [32,35]. Although, the authors do not reveal which patients were administered with the combination of the two modalities [32,35]. In parallel, Francis et al. presented the lowest RR (3.7%), even though the 4.5-year OS was 65% [37]. The latter may be due to administration with salvage EBRT only—with no use of multi-agent or multimodality regimens [37]. Regarding cohorts with I-IV stage of disease, Sears et al. obtained the best outcomes with 4.5-year DFS of 54%, OS of 51%, and RR of 23%, compared to Kuten et al. and Kamran et al., which showed higher RR [17,27,28]. The cohort of Sears et al. was administered with EBRT and/or BT after detecting locoregional recurrence. In that case, the authors provide information about patients who were explicitly administered with the combination of EBRT and boost BT, revealing a better 5-year local control of the disease (64%) compared to women administered with boost EBRT, who showed a local control of 44% [28]. Those data suggest that the combination of EBRT and BT would be the most feasible option to treat patients with an LRR of EC. Of course, it would be appropriate also to consider the other above-mentioned prognostic factors (time between surgery and recurrence, hemoglobin, recurrence on the pelvic wall, LVSI, stage of disease) to plan the treatment dose to prevent toxic reactions [51,52]. Hence, aggressive RT regimens may be applied to women who experience LRR of EC. For aggressive RT regimens, we intend at least 6000 cGy in total RT dose. In case of contraindications or severe complications due to the present management, close FU should be recommended considering the influence of prognostic factors [28,51]. On the other hand, the worst results in the literature all came from studies in which EBRT was not used as a therapeutic option, confirming the crucial role of this therapy in the management of previously untreated endometrial cancer recurrences.

The main limitation of our study is the retrospective analysis of a heterogeneous sample. Moreover, most data date back to 1980–2007. As a consequence, most data such as LVSI and histologic findings are not available in previously published studies. In addition, the scientific literature does not provide data about the use of surgery to treat locoregional recurrence in EC. Only Francis et al. employed surgery to treat three women with recurrence on the vaginal vault [37], whereas a combination of RT and surgery was used to treat eight women with vaginal vault recurrence [37]. Outcomes showed that in previously unirradiated patients treated with salvage RT, there were no statistical differences among women administered with salvage surgery or not [37]. That is why we would not routinely recommend salvage surgery for the treatment of LRR in EC. Moreover, the present review embraces a wide range of study periods in which different protocols may have been employed to treat LRRs. In parallel, the strength of our work is that it addresses the peculiar issue of LRR [53,54,55].

## 5. Conclusions

The lack of randomized clinical trials makes it difficult to understand which approach is best for the treatment of LRR. Nevertheless, in the absence of prior radiation treatment, radiation therapy appears to offer the best oncologic outcomes. The lack of extensive literature data regarding the use of chemotherapy or surgery underscores how it is necessary to tailor treatment based on the underlying condition of the disease.

## Figures and Tables

**Table 1:** Studies include ed.

Name	Country	Study Design	Period of Enrollment	FIGO Stage	No. of Participants	Treatment (Median Dose)	Mean FU * Months
Pirtoli 1980 ^ [26]	Italy	Retrospective observational monocenter study	1960–1974	-	43	External beam radiation therapy 4500–6000 rad in 5–6 weeks + boost dose 2000–3000 rad to vaginal vault	60
Kuten 1989 [27]	USA	Retrospective observational monocenter study	1959–1986	I–IV	51	Preoperative and/or postoperative external beam radiation therapy 3978 cGy and/or brachytherapy 3000 cGy	54
Sears 1994 [28]	USA	Prospective interventional monocenter study	1973–1991	I–IV	45	External beam radiation therapy 5000 cGy and/or brachytherapy 4000 cGy	89
Nag 1997 [29]	USA	Prospective interventional monocenter study	1989–1995	-	14	External beam radiation therapy 4500–5000 cGy and/or brachytherapy 3000 cGy	47
Pierga 1997 ^ [18]	France	Prospective interventional monocenter study	1992–1996	-	6	Doxorubicin, 5-fluorouracil, etoposide, and cisplatin	30
Wylie 2000 [30]	Canada	Prospective interventional monocenter study	1984–1988	I–II	58	External beam radiation therapy 8150 cGy	102
Smaniotto 2005 [15]	Italy	Prospective interventional monocenter study	1992–2003	-	30	5-fluorouracil, mitomycin C, external beam radiation therapy 4-week split course: 2340 + 2340 cGy and/or brachytherapy 2000–2500 cGy	27
Petignat 2006 [31]	Canada	Prospective interventional monocenter study	1997–2003	IB–IC	22	High-dose-rate interstitial brachytherapy 2600 cGy	32
Huh 2007 [32]	USA	Prospective interventional multicenter study	1975–2002	IA–IB–IC	69	External beam radiation therapy and/or brachytherapy	63
Attarian 2009 ^ [33]	Iran	Prospective interventional monocenter study	2004–2007	-	11	Carboplatin and Paclitaxel	30
Leslie 2013 [34]	USA	Prospective interventional monocenter study	-	-	30	Lapatinib	-
Vance 2016 ^ [35]	USA	Prospective interventional monocenter study	1989–2013	I–II	39	External beam radiation therapy 5470 cGy and/or brachytherapy 9430 cGy	56
Chapman 2017 [36]	USA	Prospective interventional monocenter study	2000–2010	I–III	30	Salvage high-dose-rate brachytherapy 6830 cGy and external beam radiation therapy in 1.8 Gy daily fractions to a total of 4500 or 5040 cGy	76
Kamran 2017 ^ [17]	USA	Retrospective case–control multicenter study	2005–2016	I–IV	66	High-dose-rate interstitial brachytherapy 7400 cGy	33
Francis 2019 ^ [37]	USA	Prospective interventional multicenter study	2000–2016	I–II	2691	Salvage external beam radiation therapy 4500 cGy	74

* Follow-Up. ^ Sub-analysis of the entire cohort.

**Table 2:** Oncological Outcomes.

Name	3Y DFS * (%)	3Y OS ° (%)	4.5Y DFS * (%)	4.5Y OS ° (%)	Recurrence Rate (%)
Pirtoli 1980 ^ [26]	-	21.0	-	16.0	-
Kuten 1989 [27]	40.0	67.5	40.0	18.0	76.4
Sears 1994 [28]	-	-	54.0	51.0	23.0
Nag 1997 [29]	-	-	85.8	67.5	30.7
Pierga 1997 ^ [18]	16.6	50.0	-	-	-
Wylie 2000 [30]	69.0	62.5	68.0	53.0	98.2
Smaniotto 2005 [15]	35.2	46.8	-	-	10.0
Petignat 2006 [31]	-	-	96.0	96.0	4.5
Huh 2007 [32]	-	-	83.0	75.0	17.0
Attarian 2009 ^ [33]	-	80.0	36.3	27.2	54.0
Leslie 2013 [34]	-	-	-	-	-
Vance 2016 ^ [35]	-	-	77.0	72.0	23.0
Chapman 2017 [36]	86.0	80.0	86.0	77.0	14.0
Kamran 2017 ^ [17]	69.0	63.0	-	-	62.0
Francis 2019 ^ [37]	-	73.0	100	65.0	3.7

* Disease-Free Survival. ° Overall Survival. ^ partial sample

**Table 3:** Site of Recurrence.

Name	Site	Local Recurrence (%)	Distant Recurrence (%)	Mixed Recurrence (%)
Pirtoli 1980 ^ [26]	-	-	-	-
Kuten 1989 [27]	Pelvis	13.7	0.0	0.0
Sears 1994 [28]	-	-	-	-
Nag 1997 [29]	Para-aortic nodes Lung Liver	0.0	35.7	0.0
Pierga 1997 ^ [18]	-	-	-	-
Wylie 2000 [30]	Vagina Pelvis	6.8	0.0	0.0
Smaniotto 2005 [15]	-	-	-	-
Petignat 2006 [31]	Para-aortic nodes Bone	0.0	4.5	0.0
Huh 2007 [32]	-	-	-	-
Attarian 2009 ^ [33]	-	-	-	-
Leslie 2013 [34]	-	-	-	-
Vance 2016 ^ [35]	-	-	-	-
Chapman 2017 [36]	-	-	-	-
Kamran 2017 ^ [17]	Iliac, inguinal, hilar, para-aortic nodes Mediastinum Peritoneal Perihepatic, presacral, prevertebral Lung Bone (L4 vertebra) Abdominal and left pelvic sidewall Vagina	10.6	21.2	4.5
Francis 2019 ^ [37]	-	-	-	-

^ partial sample.

**Table 4:** Major Complication Rate *.

Name	Complications (%)
Pirtoli 1980 ^ [26]	-
Kuten 1989 [27]	11.7
Sears 1994 [28]	-
Nag 1997 [29]	14.2
Pierga 1997 ^ [18]	0.0
Wylie 2000 [30]	-
Smaniotto 2005 [15]	13.3
Petignat 2006 [31]	68.1
Huh 2007 [32]	5.0
Attarian 2009 ^ [33]	0.0
Leslie 2013 [34]	46.6
Vance 2016 [35] ^	-
Chapman 2017 [36]	-
Kamran 2017 ^ [17]	34.0
Francis 2019 ^ [37]	-

* According to Clavien–Dindo ≥ 3. ^ partial sample

## Data Availability

Data supporting reported results can be found in the Reference List.
